# Clinic Utilization and Characteristics of Patients Accessing a Prostate Cancer Supportive Care Program’s Sexual Rehabilitation Clinic

**DOI:** 10.3390/jcm9103363

**Published:** 2020-10-20

**Authors:** Julie Wong, Luke Witherspoon, Eugenia Wu, Sara Sheikholeslami, Wen Liao, Wallace Yuen, Jenna Bentley, Christine Zarowski, Monita Sundar, Stacy Elliott, Celestia S. Higano, Ryan Flannigan

**Affiliations:** 1 Department of Urologic Sciences, University of British Columbia, Vancouver, BC V5Z 1M9, Canada; julie.w.wong@alumni.ubc.ca (J.W.); lwitherspoon@toh.ca (L.W.); wallaceyuen@alumni.ubc.ca (W.Y.); stacy.elliott@vch.ca (S.E.); 2 Department of Urology, The Ottawa Hospital, Ottawa, ON K1H 8M2, Canada; 3 Vancouver Prostate Centre, Vancouver, BC V6H 3Z6, Canada; ewu@prostatecentre.com (E.W.); ssheikoleslami@prostatecentre.com (S.S.); peggywliao@gmail.com (W.L.); jenna.bentley@vch.ca (J.B.); christine.zarowski@vch.ca (C.Z.); monita.sundar@vch.ca (M.S.); thigano@uw.edu (C.S.H.); 4 Department of Urology, University of Washington, Seattle, WA 98109-1024, USA; 5 Department of Urology, Weill Cornell Medicine, New York, NY 10065, USA

**Keywords:** sexual health, prostatic neoplasms, erectile dysfunction, urology, outpatient clinics, hospital, rehabilitation

## Abstract

Prostate cancer (PC) treatment leads to impairment of sexual function. The Prostate Cancer Supportive Care (PCSC) Program’s Sexual Rehabilitation clinic (SRC) assists patients and their partners with sexual recovery using a biopsychosocial approach to rehabilitation. This study characterizes patients seen in the SRC between July 2013–1 July 2019. Data was retrospectively abstracted from clinic records. In total, 965 patients were seen over 3391 appointments during the study period. Median age (standard deviation (SD)) was 66 years (SD = 7.1), 82.0% were partnered, yet 81.7% attended appointments alone. 88.0% were treated with surgery, 5.1% with brachytherapy, 3.7% with external beam radiation (EBRT), 1.8% with combined brachytherapy and EBRT, and 1.4% with androgen deprivation therapy. In total, 708 patients (73.4%) attended ≥1 follow-up appointment. Median time (SD) between end of prostate cancer treatment to first SRC appointment was 270 days (range 0–7766). The mean (SD) self-reported overall sexual satisfaction (extracted from International Index of Erectile Function-5 (IIEF-5)) significantly increased both with erectile aids (1.69 (SD = 1.52) to 2.26 (SD = 1.66), *p* < 0.001, *n* = 148) and without erectile aids (1.71 (SD = 1.44) to 2.35 (SD = 1.57), *p* < 0.001, *n* = 235). This study provides guidance for further investigation to refine treatment, wait-times, support, and/or resource offerings in this type of program.

## 1. Introduction

Prostate cancer (PC) is the most common malignancy affecting Canadian men, with an estimated incidence of 23,300 men in 2020 [1]. The side effects of the various treatment for prostate cancer are well documented and include impaired sexual function and sexuality, the most cited being long-lasting and distressing symptoms to patients and their partners [2,3,4,5]. Impaired sexuality has been tied to reduced overall health outcomes, relationship satisfaction, and quality of life [6,7,8].

In order to address this outcome, treating sexual health issues with a biopsychosocial approach—where medical, psychological, and interpersonal factors are all addressed—is logical [9,10,11]. This model of survivorship care allows cancer survivors and their partners to enter a multidisciplinary program that provides disease and patient-specific care plans. Despite increased interest in this treatment model, there is scant literature surrounding these programs with regard to efficacy [12].

The Vancouver Prostate Centre (VPC) and the Department of Urologic Sciences at the University of British Columbia developed the multidisciplinary Prostate Cancer Supportive Care (PCSC) Program in 2013 in order to address the complex supportive care needs of men with prostate cancer. This program is organized into seven optional modules which include: 1. An introduction to PC and treatment options; 2. Sexual rehabilitation; 3E. Exercise; 3N. Nutrition; 4. Androgen deprivation therapy; 5. Pelvic floor physiotherapy; 6. Counselling; 7. Advanced PC treatment options. The sexual rehabilitation aims to address the challenges of sexual dysfunction following PC therapy among survivors and their partners using a biopsychosocial framework. Of the seven modules, the sexual rehabilitation module is the most frequently utilized by our patients.

Herein, we characterize the patient population seen in the PCSC sexual rehabilitation program during its first 6 years of operation and report on changes in sexual satisfaction over time.

## 2. Materials and Methods

This study was approved by the University of British Columbia’s Clinical Research Ethics Board (CREB). We performed a retrospective analysis of the prospectively maintained PCSC database evaluating records from patients enrolled in the sexual rehabilitation clinic (SRC) from 17 July 2013 to 1 July 2019. Since this was a retrospective study, no additional consent needed to be obtained from patients as per CREB. All patients who had at least one consultation with the sexual rehabilitation clinic (SRC) were included for analysis. These initial consultations were almost exclusively in-person, and then were followed by in-person or telephone follow-up. Sociodemographic, diagnostic, and treatment information were gathered from the electronic medical records including age, marital status, sexual orientation, highest level of education achieved, PC Gleason score (pathological grading of the malignancy), PC clinical stage, primary PC treatment modality, and relevant medical and urologic history. Comorbidities analyzed included a history of: diabetes, hypertension, and coronary artery disease. The prospectively maintained database gathers sexual function data through semi-structured interviews and patient-reported outcome measures at initial assessment and follow-up appointments. Patients were asked to self-report sexual function using the International Index of Erectile Function (IIEF)-5, a Likert-based clinical screening tool for erectile dysfunction [13]. Characteristics of the appointment, including date of the appointment and presence of a partner were gathered through a chart review of the SRC records, which were recorded on standardized interview forms. Baseline sexual function before PC treatment was also gathered through review of initial intake appointment records. This data was de-identified and stored in a password-protected, encrypted file that was stored on a password-protected computer located at our research site. It was also held in the online PCSC database, which was password-protected with access restricted to only study co-investigators and hosted on secure local intranet infrastructure. Descriptive statistics were used to analyze patient characteristics and demographics using Microsoft Excel©. In some instances where data was not available for all patients, the number of patients available for each analysis is noted.

A physician who is subspecialized in sexual medicine leads the SRC and a registered nurse trained in sexual medicine (sexual health clinician) conducts the clinic appointments, which occur 4 days per week. The goal of the SRC is to provide education, support, and treatment pertaining to the sexual side effects of PC treatments and how sexual rehabilitation can reduce the adverse impact of PC on sexuality, sexual function, and relationships. To accomplish this, patients and their partners are invited to attend a group education session upon enrollment into the SRC. The educational session focuses on the impact of various PC treatments on sexual function, promotes understanding of the barriers to sexual adaptation post-treatment, discusses options for erectile dysfunction and other sexual issue management, and presents options for non-penetrative sexual activity. Once an educational session has been attended, individual consultations with the sexual health clinician are offered every three to six months for two years or longer, depending on the patient’s or couple’s needs. During these consultations, further counselling regarding sexual adaptation, penile rehabilitation, and options for erectile dysfunction (i.e., phosphodiesterase 5 inhibitors, intra cavernosal injections, vacuum therapy, and referral for penile implant surgery) as well as other sexual issues management are discussed (i.e., disorders in arousal, climacturia or arousal incontinence, dysorgasmia, anorgasmia). If further medical or surgical intervention is warranted, the patient is referred to the SRC physician. Patients and their partners are also provided links to educational videos and handouts, which provide more detailed information regarding: sexual adaptation, penile rehabilitation, use of phosphodiesterase 5 inhibitors, performing penile injection therapy, vacuum erection therapy, managing climacturia and arousal incontinence, penile implant therapy, intimacy tips, managing orgasmic dysfunction following prostate cancer therapy, sexual function for single and gay men following prostate cancer therapy. Couples who have been evaluated within the SRC are invited to attend a workshop on intimacy facilitated by two sexual health clinicians. The intimacy workshop is offered quarterly. In summary, after the initial group education session, patients then attend consultations with the sexual health clinician (a specially trained registered nurse), with referral to the SRC physician if deemed necessary.

Patients can be referred to the SRC by any physician, be it their urologist, radiation oncologist, medical oncologist, or primary care physician. Patients seen at the SRC before prostate cancer treatment were removed from the analysis of time between primary PC treatment and first SRC intake appointment and were excluded from the pre/post analysis of mean self-reported overall sexual satisfaction, unless they had a self-reported score from their first SRC appointment after PC treatment.

Annual total numbers of radical prostatectomies (RP) performed at VPC from 2013–2018 were retrieved in order to calculate the proportion of patients that were referred to the SRC. Similarly, total numbers of curative intent brachytherapy patients and curative intent external beam radiation therapy (EBRT) from 2013–2018 at the BC Cancer Agency were retrieved for similar calculations. Since annual numbers were only available, an analysis of treatment numbers of 2019 was omitted given incomplete annual data. Mean self-reported overall sexual satisfaction was analyzed for patients comparing their initial scores after therapy and their scores at the end of program enrollment using parametric paired t-tests.

## 3. Results

SRC data from 965 patients and 3391 appointments were analyzed. The median patient age was 66 years, with a range of 42–92 years and a standard deviation (SD) of 7.1 years. The majority (80.8%) had education beyond a high school diploma. Assessing pre-existing comorbidities, 9.5% of patients had diabetes, 24.5% of patients had hypertension, and 11.2% of patients had coronary artery disease. With regard to baseline sexual function before therapy, the majority (74.8%) reported being sexually active with 29.6% taking phosphodiesterase type 5 inhibitors (PDE5 inhibitors) as needed and 3.1% regularly using some sort of sexual aid (PDE5 inhibitors, vacuum erectile device (VED), or intracavernosal injection (ICI)) (Table 1). Most patients (82.0%) were partnered (reporting either as married, common-law/co-habitating, or partnered), with 95.2% identifying as heterosexual. Attendance with a partner for at least one appointment was noted in 35.6% of patients, but the majority of appointments were attended alone (81.7%). The patient was most commonly accompanied by a partner at the initial appointment (33.0%) compared to 12.4% of follow-up appointments (Table 2).

Of the total, 708 patients (73.4%) attended at least 1 follow-up appointment. Of this subset, the median number of follow-up appointments was 3 (range 1–14). Of this subset, the median number of days enrolled in the program was 406 days (range 10–2015).

**Figure 1:**
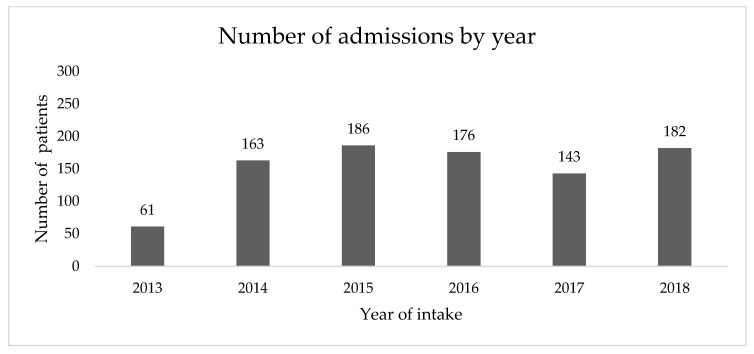
Number of admissions to the Vancouver Prostate Cancer Supportive Care Program’s Sexual Rehabilitation clinic by year.

**Figure 2:**
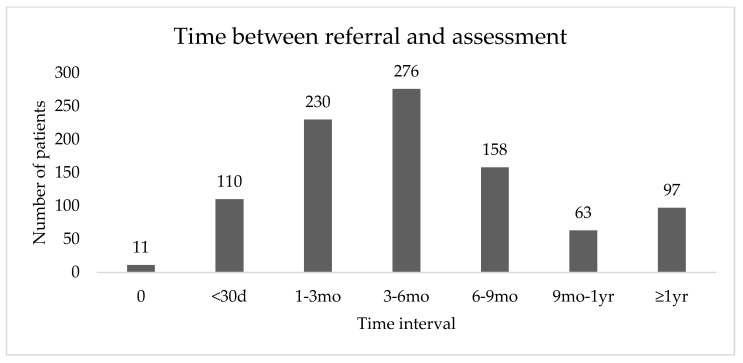
Time from referral to the Vancouver Prostate Cancer Supportive Care Program’s Sexual Rehabilitation clinic to initial assessment at the clinic.

Annual enrollment in the SRC program ranged from 143–182 new patients (Figure 1). Since the study period ended July 1 2019, annual enrollment for 2019 was not calculated. The median time interval between referral to the program and first appointment in the SRC was 126.5 days (range 0–1769) (Figure 2). From 2013–Jun 30 2018, the median time between referral and first appointment was 124 days (range 0–1539). From July 1 2018–July 1 2019, this time was decreased to 105.5 days (range 1–1769).

Of the 899 patients with known primary PC treatment modality, 88.0% were treated with surgery, 5.1% brachytherapy, 3.7% EBRT, and 1.4% primary androgen deprivation therapy (ADT). As compared to the total number of cases treated by VPC (surgery) or BC Cancer Agency (radiation) per year, SRC patients make up 15–35% of all surgery patients and 0–2% of all radiation patients (Table 3). For the 855 patients whose first SRC appointment occurred post-PC treatment, the median time between the end of primary PC treatment to first SRC appointment was 270 days, with a SD of 1042.6 days (range 0–7766). From 2013–30 June 2018, the median time between the end of prostate cancer treatment to first SRC appointment was 281 days with a SD of 1054.3 days (range 0–7766). From 1 July 2018–1 July 2019, this time was decreased to 175 days with a SD of 923.8 days (range 42–6537). 855 patients were included in the analysis of the time interval between primary PC treatment and first appointment at the SRC, with 18 patients being excluded as their first SRC appointment occurred prior to PC treatment and patients being excluded as their modality of primary PC treatment was unknown. The median time interval was 270 days, with 299 (35.0%) patients having their first SRC appointment 0–6 months after treatment. When analyzing this time interval by treatment modality, an additional 36 patients were excluded as their modality of primary PC treatment was unknown. The majority of EBRT (50%) and brachytherapy (58.8%) patients were seen more than 24 months after their PC treatment (Table 4).

From 2017 onwards, self-reported erectile function and sexual satisfaction scores were gathered. Over this period of enrollment in the SRC clinic, mean self-reported overall sexual satisfaction (IIEF-5) significantly increased both with and without erectile aids. Out of 148 patients who self-reported their performance with erectile aids, mean sexual satisfaction (mean, SD) significantly increased from 1.69 (SD 1.52) to 2.26 (SD 1.66) at last follow up (*p* < 0.001). Of 235 patients who self-reported performance without erectile aids, a significant increase was also observed when comparing between baseline and last follow up, with a baseline mean (SD) score of 1.71 (SD 1.44) increasing to a mean (SD) score of 2.35 (SD 1.57) at last follow up (*p* < 0.001).

## 4. Discussion

### 4.1. Main Findings

This study provides a descriptive analysis of a large population of patients treated for prostate cancer who were evaluated in a SRC that focuses on a biopsychosocial approach to therapy. To our knowledge, this is the largest collection of patients reported in the literature. Our SRC enrolled 143–182 new patients annually and saw a total of 965 new patients over a 6-year period.

Our patient population appeared to have a higher rate of ED as compared to the general population with 29.6% of patients reporting use of PDE5 inhibitors at least occasionally as their pre-treatment baseline. The general population has an estimated prevalence of ED of 18.4% [14], with studies showing 34.1% of patients with ED had tried PDE5 inhibitors at least once [15]. This is expected, as our patient population is older than the general population and thus is expected to have a higher baseline rate of ED.

Of the 73% of patients in the program who returned for at least 1 follow up appointment, the median number of visits was 3, suggesting that patients found the program useful. Although previous recommendations have suggested that the optimal duration for a sexual rehabilitation program is 2 years, the median number of days (406) that men spent in the SRC suggests that a shorter duration may also be beneficial [16].

There was an imbalance in the proportion of men who were treated with surgery as opposed to radiation therapy in our program. This is likely due to the fact that the SRC is co-located in a urology clinic and that men are more impacted immediately after surgery than they are with radiation. Patients who receive radiation therapy are treated in another facility. Although the institutions are only a few blocks away from each other, there is less awareness of the SRC in the other facility. In addition, patients treated with primary radiation therapy may not experience sexual health symptoms until much later after completing therapy consistent with a higher proportion of radiation patients referred to the SRC over two years from completing therapy. At that time, most patients have returned to their primary care physicians who may not be aware of the SRC or, because the onset of symptoms is more gradual, patients may be more accepting of these changes and less likely to seek help.

### 4.2. Interpretation and Comparison to other Studies

The majority of the patients in the SRC were heterosexual, with 4.8% identifying otherwise, including one transgender patient. The literature has shown that gay and bisexual men also suffer negative impacts on their sexual functioning after PC treatment and often face difficulties with their sexual rehabilitation journey [17]. It is unclear if the low enrollment of LGBQT+ (lesbian, gay, bisexual, queer, transgender, plus) patients in our program reflects an inherent bias in our programing, making it less appealing/useful to this population, or if this rate of enrollment is reflective of the proportion of LGBQT+ PC patients in British Columbia. Nevertheless, it is important to include LGBQT+ patients in our SRC and to tailor sexual rehabilitation plans to the unique needs of these patients.

Self-reported sexual satisfaction increased at the end of SRC treatment compared to the initial assessment, both with and without erectile aids. This can partially be attributed to the known improvement in erectile function over time following PC treatment. However, the majority of sexual recovery occurs in the first 18 months after treatment, with the most significant increase happening in the first 6 months [18,19]. Median IIEF-5 scores have been shown to increase 13–17 points from 6 weeks post-operatively to 18 months post-operatively for patients treated with RP [18], but, to our knowledge, no studies exist looking specifically at increase of the IIEF-5 subset score of sexual satisfaction over time in PC survivors without intervention. At present, it is not clear whether this improvement was related to the passage of time, the SRC program, or both. Additional patient-reported outcome scores, further standardized follow-up, and potential randomized controlled trials are necessary to better understand the contribution of biopsychosocial programs such as the SRC to improve patient outcomes.

Previous literature has shown that 3 months post-treatment or earlier, is considered the preferable time to begin sexual rehabilitation [20]. Initially, the program was structured with the goal of seeing patients for initial intake at 12 weeks post-treatment, but now the goal is to perform an initial intake assessment at 6 weeks post-treatment. Only 35% of our patients were in the 0–6 months post treatment time frame. We have instituted changes to our procedures which will shorten the time to enter the SRC program including increased promotion of the program, restructuring clinical appointments and documentation, and automated referrals from urologists at our center. Following implementation of these changes the median time from prostate cancer treatment to SRC intake decreased to 175 days (SD = 923.8) (range 42–6537) with a similar decrease in median time from referral to SRC intake to 105.5 days (SD = 264.0) (range 1–1769). These wait times are generally favorable to the widely ranging wait times for erectile dysfunction among urologists in the province of British Columbia, with subspecialty sexual medicine waitlists in the order of 6–12 months. Nonetheless, additional collaboration with radiation oncology colleagues and primary care physicians is necessary to improve awareness of the SRC program and encourage referral to interested patients and couples.

### 4.3. Strengths and Limitations

Although 965 patients attended an initial intake appointment at the SRC, only 708 patients (73.4%) had a follow-up appointment. Further investigation into this attrition rate should be conducted to identify contributing factors. When looking at the literature for other rehabilitation clinics, namely cardiac and stroke rehabilitation, follow up attendance rates appear similar at 80% [21,22].

Although the majority of our patients were in a relationship, most of the partners did not accompany the patient after the initial SRC appointment. A factor that could account for this observation is that many of the follow-up appointments were done via telephone. A script inviting the partner to participate on speaker phone or virtual health platform may optimize couple participation.

As a descriptive study reliant on patient-reported data, there was, as expected, some missing data. Furthermore, the self-reported sexual satisfaction data forms were not incorporated into clinic visits until 2017, so pre-post comparisons were not available prior to that time. This is a single-center study so the results may not be generalizable to other clinical settings.

### 4.4. Conclusions and Implications for Practice and Future Research

Sexual side effects from PC treatment can be long-lasting and have significant adverse effects on quality of life. This study describes the clinic experience in our SRC, one of the few multidisciplinary clinics in Canada that supports PC survivors through sexual rehabilitation. This research provides guidance for further investigation to refine treatment, wait-times, support, and/or resource offerings in this type of program. We are currently conducting further research into the treatment recommendations offered to patients at the SRC and treatment outcomes. The patient demographics and clinic utilization described here can contribute to the growing data regarding sexual rehabilitation for PC patients as programs and clinicians continue to provide individualized care and support to patients and their partners throughout their journey with PC.

## Figures and Tables

**Table 1:** Demographic data of patient attending the Prostate Cancer Supportive Care (PCSC) Program between 2012–2019.

		Available Data
Total Patients	965	*n* = 965
Age (median years, range, standard deviation (SD))	66 (42–92) SD = 7.1	*n* = 965
Ethnicity		*n* = 377
African/African-American/Caribbean (*n*, % of total)	11 (2.9%)	
Caucasian (*n*, % of total)	283 (75.0%)	
East Asian (*n*, % of total)	44 (11.7%)	
Latino/Hispanic (*n*, % of total)	6 (1.6%)	
Middle Eastern (*n*, % of total)	5 (1.3%)	
Mixed (*n*, % of total)	3 (0.8%)	
South Asian (*n*, % of total)	21 (5.6%)	
Other/Prefer not to answer (*n*, % of total)	4 (1.1%)	
Education (Highest Completed)		*n* = 498
High school (*n*, % of total)	96 (19.2%)	
Apprenticeship/non-university diploma (*n*, % of total)	79 (15.9%)	
University undergraduate (*n*, % of total)	197 (39.6%)	
Graduate (*n*, % of total)	125 (25.3%)	
Comorbidities		*n* = 860
Diabetes (*n*, % of total)	82 (9.5%)	
Hypertension (*n*, % of total)	211 (24.5%)	
Coronary artery disease (*n*, % of total)	96 (11.2%)	
Gleason Score		*n* = 825
<6 (*n*, % of total)	3 (0.4%)	
6 (*n*, % of total)	211 (25.6%)	
3 + 4 = 7 (*n*, % of total)	281 (34.1%)	
4 + 3 = 7 (*n*, % of total)	116 (14.1%)	
8 (*n*, % of total)	102 (12.4%)	
9+ (*n*, % of total)	112 (13.6%)	
Primary Treatment Type		*n* = 899
Prostatectomy (*n*, % of total)	791 (88.0%)	
Brachytherapy (*n*, % of total)	46 (5.1%)	
External beam radiation therapy (EBRT) (*n*, % of total)	33 (3.7%)	
EBRT + brachytherapy (*n*, % of total)	16 (1.8%)	
Androgen deprivation therapy (*n*, % of total)	13 (1.4%)	
Baseline Sexual Function Pre-Prostate Cancer		
Sexually active (*n*, % of total)	718 (74.3%)	
Phosphodiesterase type 5 inhibitor (PDE5 inhibitor) as needed (*n*, % of total)	286 (29.6%)	
PDE5 inhibitor daily (*n*, % of total)	15 (1.6%)	
Vacuum erectile device (VED) (*n*, % of total)	5 (0.5%)	
Intracavernosal injection (ICI) (*n*, % of total)	10 (1.0%)	

**Table 2:** Marital status, sexual orientation, and partner attendance at Prostate Cancer Supportive Care (PCSC) Program appointments.

		Available Data
Total Patients	965	*n* = 965
Marital Status		*n =* 672
Married (*n*, % of total)	507 (75.5%)	
Common-law/co-habitating (*n*, % of total)	21 (3.1%)	
Partnered (*n*, % of total)	23 (3.4%)	
Divorced (*n*, % of total)	20 (3.0%)	
Separated (*n*, % of total)	21 (3.1%)	
Widowed (*n*, % of total)	15 (2.2%)	
Single (*n*, % of total)	63 (9.4%)	
Other (*n*, % of total)	2 (0.3%)	
Sexual Orientation		*n =* 461
Heterosexual (*n*, % of total)	439 (95.2%)	
Homosexual (*n*, % of total)	19 (4.1%)	
Bisexual (*n*, % of total)	3 (0.7%)	
Partner Attendance at Appointments		
Total appointments with a partner present (*n*, % of total)	620 (18.3%)	*n =* 3391
Initial appointments with a partner present (*n*, % of total)	318 (33.0%)	*n =* 965
Follow-up appointments with a partner present (*n*, % of total)	302 (12.4%)	*n =* 2426
Patients with partners present at ≥1 appointment (*n*, % of total)	346 (35.8%)	*n =* 965
Number of appointments patients attended with a partner (median, interquartile range)	0 (1)	
Number of appointments patients attended with a partner, of those who had ever had partners present (median, interquartile range)	1 (0)	

**Table 3:** Patients enrolled in the Prostate Cancer Supportive Care (PCSC) Program by primary treatment type by year.

Primary Treatment Type by Year		Total Treated
2012	Radical prostatectomy (RP) (*n*, % of total treated)	42 (15%)
	Brachytherapy (*n*, % of total treated)	3 (2%)
	External beam radiation therapy (EBRT) (*n*, % of total treated)	3 (1%)
2013	RP (*n*, % of total treated)	81 (30%)
	Brachytherapy (*n*, % of total treated)	3 (2%)
	EBRT (*n*, % of total treated)	1 (0%)
2014	RP (*n*, % of total treated)	89 (35%)
	Brachytherapy (*n*, % of total treated)	1 (1%)
	EBRT (*n*, % of total treated)	2 (1%)
2015	RP (*n*, % of total treated)	71 (29%)
	Brachytherapy (*n*, % of total treated)	0 (0%)
	EBRT (*n*, % of total treated)	4 (2%)
2016	RP (*n*, % of total treated)	67 (28%)
	Brachytherapy (*n*, % of total treated)	2 (2%)
	EBRT (*n*, % of total treated)	0 (0%)
2017	RP (*n*, % of total treated)	75 (27%)
	Brachytherapy (*n*, % of total treated)	1 (1%)
	EBRT (*n*, % of total treated)	0 (0%)
2018	RP (*n*, % of total treated)	82 (27%)
	Brachytherapy (*n*, % of total treated)	4 (3%)
	EBRT (*n*, % of total treated)	1 (0%)

**Table 4:** Time between prostate cancer (PC) treatment and Prostate Cancer Supportive Care (PCSC) Program Sexual Rehabilitation Clinic (SRC) intake appointment.

Time between Prostate Cancer (PC) Treatment and Clinic Intake (Months)		Median
Overall (days)		270
<0 (% of total)		18 (2.1%)
	Radical prostatectomy (RP) (*n*, % of treatment modality)	10 (1.3%)
	Brachytherapy (*n*, % of treatment modality)	0 (0%)
	External beam radiation therapy (EBRT) (*n*, % of treatment modality)	0 (0%)
0–6 (% of total)		299 (34.2%)
	RP (*n*, % of treatment modality)	273 (36.2%)
	Brachytherapy (*n*, % of treatment modality)	8 (15.7%)
	EBRT (*n*, % of treatment modality)	7 (29.2%)
6–12 (% of total)		207 (23.7%)
	RP (*n*, % of treatment modality)	188 (24.9%)
	Brachytherapy (*n*, % of treatment modality)	6 (11.8%)
	EBRT (*n*, % of treatment modality)	2 (8.3%)
12–24 (% of total)		145 (16.6%)
	RP (*n*, % of treatment modality)	133 (17.6%)
	Brachytherapy (*n*, % of treatment modality)	7 (13.7%)
	EBRT (*n*, % of treatment modality)	3 (12.5%)
>24 (% of total)		204 (23.4%)
	RP (*n*, % of treatment modality)	150 (19.9%)
	Brachytherapy (*n*, % of treatment modality)	30 (58.8%)
	EBRT (*n*, % of treatment modality)	12 (50%)
